# Organisational Impact of Remote Patient Monitoring for Heart Failure Management: A Survey of 28 Cardiology Departments and Community Practices in France

**DOI:** 10.3390/ijerph23070933

**Published:** 2026-07-21

**Authors:** Benoit Lequeux, Thierry Garban

**Affiliations:** 1 CHU Poitiers, 86000 Poitiers, France; 2 Cabinet de Cardiologie, 44000 Nantes, France

**Keywords:** remote patient monitoring, heart failure, organisational impact, health technology assessment, telemonitoring, task delegation, France

## Abstract

**Highlights:**

**Public health relevance—How does this work relate to a public health issue?**

- Heart failure affects over 64 million people worldwide and represents a leading cause of hospitalisation, driving demand for innovative care models.
- RPM offers a scalable, evidence-based approach to reducing cardiac decompensation delays and hospital readmissions.

**Public health significance—Why is this work of significance to public health?**

- This is one of the first surveys to assess the organisational impact of RPM across both hospital and community cardiology settings in France.
- The findings demonstrate that RPM drives significant structural reorganisation, including task delegation (89.3%) and dedicated team formation (96.4%).

**Public health implications—What are the key implications or messages for practitioners, policy makers and/or researchers in public health?**

- As RPM transitions to standard reimbursement in France, addressing training gaps (only 53.6% trained) and interprofessional coordination is essential
- Investment in standardised digital coordination platforms between hospital and community settings is a priority for maximising the public health benefits of RPM.

**Abstract:**

Background: Remote patient monitoring (RPM) for chronic heart failure (CHF) management has demonstrated clinical and economic benefits. However, data on its organisational impact remain limited, particularly in the French healthcare context following the integration of RPM into standard care pathways. The objective of this study was to describe the organisational impact of RPM for CHF management from the perspective of healthcare professionals across diverse practice settings in France. Methods: A cross-sectional survey was conducted among 28 French cardiology departments and community practices (14 CHG, 9 CHU, 4 community practices, 1 private clinic) actively using RPM for CHF management. The questionnaire assessed organisational changes across five domains: care process modifications, human resources allocation, professional training and task delegation, coordination with community-based physicians, and perceived impact on patient quality of life and working conditions. Results: Nearly all structures (96.4%) had established a dedicated RPM team (mean size: 4.8 ± 2.3 professionals). A reduction in time between cardiac decompensation and medical care was reported by 96.4% of respondents. Three-quarters (75.0%) had implemented specific consultations for emergency alerts, and 57.1% had established direct admission protocols bypassing emergency departments. Task delegation was widespread (89.3%), primarily involving alert monitoring (100%), patient inclusion (92.0%), and patient contact (84.0%). However, only 53.6% of professionals had received specific training. The perceived impact on patient quality of life was unanimously positive (64.3% very positive, 35.7% positive). Working conditions were perceived as stable by 64.3% and improved by 32.1%. The main advantages cited were reduction in hospitalisations (89.3%) and improved patient communication (85.7%). The primary area for improvement was interprofessional coordination (53.6%). Conclusions: RPM implementation has driven substantial organisational restructuring across French cardiology departments and community practices. While dedicated teams and task delegation are now standard, challenges remain in professional training, coordination with community physicians, and dedicated time allocation. These findings provide an updated organisational assessment that complements prior clinical and economic evaluations.

## 1. Introduction

Heart failure (HF) is a major public health challenge, affecting an estimated 64.3 million people worldwide and representing a leading cause of hospitalisation in patients over 65 years of age [1,2]. In France, chronic heart failure (CHF) accounts for approximately 165,000 hospitalisations annually, with readmission rates exceeding 25% within 30 days of discharge [3]. The growing burden of CHF has prompted the development of innovative care strategies, among which remote patient monitoring (RPM) has emerged as a promising approach.

RPM enables continuous or periodic assessment of patients’ clinical parameters outside the hospital setting, facilitating early detection of decompensation and timely therapeutic intervention [4]. Recent meta-analyses have demonstrated that RPM reduces HF-related hospitalisations (RR = 0.80, 95% CI: 0.77–0.84) and improves quality of life (SMD = 0.23, 95% CI: 0.20–0.26) [5]. The 2021 ESC Guidelines for heart failure management have acknowledged the potential role of RPM in improving outcomes, particularly for patients with a recent hospitalisation [2].

In France, RPM for CHF was initially deployed within the ETAPES programme (Expérimentations de Télémédecine pour l’Amélioration des Parcours En Santé), which provided a regulatory framework for evaluating RPM devices [6]. Since 2023, RPM has been integrated into the standard reimbursement pathway (droit commun), marking a transition from experimental to routine practice. The French National Authority for Health (Haute Autorité de Santé, HAS) published a guide in 2020 defining organisational impact as ‘an effect or consequence of the health technology on the characteristics and functioning of an organisation involved in the care process’ and establishing criteria for its assessment [7].

While clinical and economic evaluations of RPM have been extensively documented [8,9], the organisational impact has been comparatively understudied. A seminal survey by Alami et al. (2023) described the organisational impact of a specific RPM system (Chronic Care ConnectTM) across 29 French cardiology departments [10]. However, as RPM has matured and expanded to include community-based practices, an updated assessment across diverse care settings is warranted.

The objective of the present study was to describe the organisational impact of RPM for CHF management from the perspective of healthcare professionals in 28 French cardiology departments and community practices, using the HAS organisational impact framework as the analytical structure.

## 2. Materials and Methods

### 2.1. Survey Design

A cross-sectional survey was conducted among French cardiology departments and community cardiology practices actively using RPM for CHF management. An online questionnaire was designed to capture the organisational impact across the domains defined by the HAS framework for health technology assessment [7]. The questionnaire comprised 25 items covering: (1) the care process (dedicated teams, specific consultations, direct admission protocols, time to care); (2) human resources and equipment; (3) professional training and task delegation; (4) coordination with community-based physicians; and (5) perceived impact on patient quality of life and professional working conditions. The survey was administered as a single, one-time cross-sectional data collection conducted between September and December 2025; the questionnaire was not repeated longitudinally.

### 2.2. Organisational Impact Framework

The analysis was structured according to the HAS organisational impact criteria [7], which define three macro-criteria: (1) impacts on the care process, including timing, content, human resources, and equipment; (2) impacts on the abilities and skills required of stakeholders, including training, task delegation, and information sharing; and (3) impacts on coordination between stakeholders, including hospital–community integration and perceived outcomes. This framework was previously applied by Alami et al. [10] and allows for comparison with earlier findings.

### 2.3. Participants

The questionnaire was distributed to 28 structures actively using RPM for CHF management. In each structure, the questionnaire was designed to be completed collectively by the RPM team. The sample included 14 centres hospitaliers généraux (CHG, 50.0%), 9 centres hospitaliers universitaires (CHU, 32.1%), 4 community cardiology practices (cabinets de ville, 14.3%), and 1 private clinic (3.6%). The inclusion of community practices represents a notable extension compared to the exclusively hospital-based sample in Alami et al. [10].

Participating structures were identified from the base of French establishments using the Optified Self™ telemonitoring platform (Beys Health Solutions, Paris, France) for the management of heart failure, and all eligible structures were invited to take part in the survey; enrolment thus constituted a convenience sample rather than a randomly drawn one. The number of patients monitored per structure was not collected in this survey, whose objective was to assess the organisation of structures implementing telemonitoring rather than their level of activity; we acknowledge that this information would have helped to better characterise the experience of the participating centres, and it is now noted as a limitation in the Section 4. The urban versus rural distribution of the participating structures was not systematically recorded and therefore cannot be reported.

### 2.4. Statistical Analysis

All statistical analyses were descriptive. Categorical variables were expressed as frequencies and percentages. Continuous variables were expressed as means, standard deviations, and medians. Multi-response items were analysed as proportions of total respondents. Subgroup analyses were conducted by type of structure (CHU, CHG, community practice). Analyses were performed using standard spreadsheet software. Given the descriptive nature and small sample size, no formal hypothesis testing was applied.

## 3. Results

### 3.1. Characteristics of Participating Structures

All 28 structures (100% response rate) completed the survey. The distribution by type of structure is presented in Table 1. The mean dedicated team size was 4.8 ± 2.3 professionals (median: 5.0; range: 2–11), with data available for 25 structures.

### 3.2. Impact on the Care Process (Macro-Criterion 1)

#### 3.2.1. Dedicated Teams and Consultations

Nearly all responding structures (27/28, 96.4%) had established a team specifically dedicated to RPM. This proportion is higher than the 83% reported by Alami et al. [10], suggesting progressive standardisation of this organisational feature. Three-quarters of the structures (21/28, 75.0%) had implemented specific consultations for patients receiving emergency alerts through the RPM device, compared with 55% in the Alami et al. study [10].

#### 3.2.2. Direct Admission Protocols

Sixteen structures (57.1%) had established a protocol for direct admission, bypassing emergency department attendance. This proportion was higher among hospital-based structures: 71.4% of CHG and 66.7% of CHU, compared with 0% of community practices—which is consistent with their ambulatory care role. In the Alami et al. study, 86% of departments reported direct admission, though their sample was exclusively hospital-based [10].

#### 3.2.3. Reduction in Time to Care

The vast majority of respondents (27/28, 96.4%) reported a reduction in the time between cardiac decompensation and the initiation of medical care (Table 2). This is consistent with the findings of Alami et al., where 72% ‘absolutely agreed’ and 24% ‘mostly agreed’ with this statement [10]. Among those who could quantify this reduction, reported times ranged from ‘immediate’ to ‘one to two weeks’, with the most common response being ‘one to two days’. This finding aligns with meta-analytic evidence showing that RPM significantly reduces time to clinical intervention [5].

### 3.3. Impact on Skills and Task Delegation (Macro-Criterion 2)

#### 3.3.1. Healthcare Professionals Involved

Cardiologists were involved in RPM in 27 of 28 structures (96.4%), followed by nurses (75.0%), specialist nurses (ISPIC, 50.0%), advanced practice nurses (IPA, 25.0%), dietitians (25.0%), and psychologists (10.7%). Compared with Alami et al. [10], the composition of RPM teams has diversified, with greater involvement of IPA and allied health professionals, reflecting the evolution of care models toward multidisciplinary approaches.

#### 3.3.2. Training

Just over half of the structures (15/28, 53.6%) reported that their professionals had received specific training for RPM. This is lower than the 66% reported by Alami et al. [10], which may reflect the expansion of RPM to settings with fewer training resources, rather than a decline in training provision per se. Training hours varied considerably, from 1 to 3 h for basic operational training to over 150 h for professionals completing a university diploma in heart failure (DIU IC) combined with a cooperation protocol (protocole de coopération).

#### 3.3.3. Task Delegation

Task delegation from cardiologists to nurses was implemented in 25 of 28 structures (89.3%), compared with 52% in Alami et al. [10]. This substantial increase reflects the maturation of RPM programmes and the broader adoption of cooperation protocols. The most frequently delegated tasks were: alert monitoring (100%), patient inclusion (92.0%), patient contact (84.0%), and treatment adjustment (48.0%) (Table 3). The delegation of treatment adjustment, while still below 50%, represents an important extension of nursing competencies that may further expand as cooperation protocols become more widespread. In this study, treatment adjustment denotes adaptations of pharmacological therapy carried out by nurses within a validated cooperation protocol (protocole de coopération) and under the cardiologist’s supervision—principally titration of guideline-directed heart-failure therapy or adjustment of diuretics according to predefined protocols—rather than referral of the patient for an outpatient consultation. Our questionnaire did not explore the reasons for the absence of task delegation, so the organisational factors that led three structures (10.7%) to retain a model without delegation cannot be established with certainty; several explanations are plausible, including local organisation, human-resource constraints, or the absence of a cooperation protocol. More generally, in France the delegation of certain medical acts to nurses is governed by formally authorised cooperation protocols, which constitute the regulatory and medico-legal framework enabling nurses to perform delegated acts under a physician’s responsibility.

### 3.4. Coordination with Community-Based Physicians (Macro-Criterion 3)

Information from RPM was shared with community physicians primarily through letters (54.2%), email (54.2%), and telephone (50.0%), with only one structure using a dedicated digital platform (4.2%). One structure reported no information sharing with community physicians. Notably, only 10 of 28 structures (35.7%) perceived an improvement in coordination with community physicians as a result of RPM. This finding is consistent with Alami et al. [10], where coordination with ambulatory care was identified as a key challenge. The limited use of digital platforms for information sharing may contribute to this gap, as highlighted by studies on integrated digital solutions in care coordination [4,11].

### 3.5. Equipment and Infrastructure

Eighteen structures (64.3%) had invested in specific equipment for RPM management. Among those, the most common investments were: dedicated email addresses (77.8%), dedicated telephone lines (72.2%), RPM rooms (55.6%), dedicated consultation offices (38.9%), and dedicated computers or screens (22.2%). These findings are consistent with Alami et al. [10], who reported that RPM did not require substantial capital investment, relying primarily on existing computing infrastructure supplemented by dedicated communication tools.

### 3.6. Perceived Impact on Quality of Life and Working Conditions

The perceived impact of RPM on patients’ quality of life was unanimously positive, with 64.3% rating it as ‘very positive’ and 35.7% as ‘positive’. No respondent perceived a neutral or negative impact (Table 4). These results are consistent with patient-reported data from the Satelia^®^ Cardio programme, where 87% of CHF patients expressed satisfaction with RPM and 45% of digitally literate patients reported improved quality of life [12]. It should be emphasised that quality of life was not measured with a validated patient-reported outcome instrument; the ratings reflect the subjective perception of the responding RPM teams regarding the impact of RPM on their patients. The same applies to the perceived advantages and areas for improvement reported in Section 3.7, which represent professionals’ subjective assessments rather than objectively measured endpoints such as hospitalisation counts or emergency-department visits.

Regarding working conditions, 32.1% of respondents reported an improvement, 64.3% described stable conditions, and one respondent (3.6%, a community practice) reported a degradation. The predominance of ‘stable’ responses reflects the dual nature of RPM: while it improves care processes, it also creates additional workload related to alert management and administrative tasks, as previously described [10,13].

### 3.7. Perceived Advantages and Areas for Improvement

The principal advantages perceived by respondents (Table 5) were: reduction in hospitalisations (89.3%), improved patient–clinician communication (85.7%), reduced time to care (75.0%), and reduced duration of hospitalisation (67.9%). Several respondents additionally mentioned improved patient autonomy, better treatment adherence, and enhanced patient confidence in hospital-based care.

The main areas for improvement identified were: interprofessional coordination (53.6%), professional training (28.6%), human resources (21.4%), and dedicated time allocation for RPM activities (7.1%). The prominence of coordination as the primary concern is consistent with the limited improvement perceived in hospital–community coordination (35.7%) and underscores the need for integrated digital platforms and structured collaboration pathways [4,11].

## 4. Discussion

The present survey provides an updated description of the organisational impact of RPM for CHF management across 28 French cardiology departments and community practices. By extending the analytical scope to include community-based practices alongside hospital departments, this study offers a more comprehensive picture of how RPM is reshaping CHF care organisation in France.

Our results demonstrate significant organisational maturation compared to the findings of Alami et al. [10]. The proportion of structures with a dedicated RPM team has increased from 83% to 96.4%, specific consultations for alerts from 55% to 75.0%, and task delegation from 52% to 89.3%. These trends suggest that as RPM programmes mature, the associated organisational structures become increasingly standardised and embedded within routine care processes.

A particularly noteworthy finding is the near-universal task delegation (89.3%), with alert monitoring delegated in 100% of cases. This reflects the evolving role of nurses—particularly ISPIC and IPA—in CHF management, consistent with broader trends toward collaborative practice protocols in French cardiology [14]. The delegation of treatment adjustment, while still reported by less than half of the structures (48.0%), represents an important extension of nursing competencies that may further expand as cooperation protocols become more widespread.

Despite these advances, several challenges persist. First, only 53.6% of professionals had received specific training, suggesting that the pace of organisational restructuring has outstripped the provision of formal training. This gap represents a patient safety concern that warrants attention from professional bodies and programme coordinators. Second, coordination with community physicians remains limited: only 35.7% of respondents perceived an improvement, and information sharing still relies predominantly on traditional methods (letters, telephone). The minimal use of digital platforms (4.2%) contrasts with the technological sophistication of the monitoring systems themselves, highlighting an important gap in care coordination infrastructure.

The inclusion of community practices in our sample reveals structural differences in RPM implementation between hospital and ambulatory settings. Community practices had no direct admission protocols (0% vs. 67–71% in hospitals), reflecting their different role in the care pathway. However, they reported similar rates of dedicated teams and task delegation, suggesting that RPM organisational principles are transferable across practice settings, even when specific modalities differ.

The unanimously positive perception of RPM’s impact on patient quality of life (100% positive or very positive) is consistent with patient-reported data from the Satelia^®^ Cardio programme [12] and with meta-analytic evidence demonstrating improved quality of life scores (SMD = 0.23) [5]. The predominantly stable impact on working conditions (64.3%) with a minority reporting improvement (32.1%) reflects the documented tension between improved care processes and the additional administrative burden associated with RPM [10,13].

This study has several limitations that should be acknowledged. First, the survey design is cross-sectional and declarative, relying on professionals’ self-reported perceptions rather than objective organisational metrics. Second, the sample size (N = 28) limits the statistical power for subgroup analyses and generalisability. Third, the questionnaire did not capture quantitative data on patient volumes, alert management time, or economic outcomes. Finally, the study evaluated a single telemonitoring platform (Optified Self™); while this ensures a consistent monitoring modality across respondents, it may limit the generalisability of the findings to other RPM solutions. Future multicentre studies with standardised metrics and longitudinal designs are needed to address these limitations. Accordingly, the reported effects on time to care, hospitalisations, and quality of life should be interpreted as respondents’ appraisals rather than as independently verified operational outcomes; they remain informative for characterising organisational change and are transparently framed as perceptions throughout the manuscript.

Future research should combine quantitative health data (hospitalisation rates, lengths of stay, emergency department visits) with organisational assessments to establish the relationship between organisational models and clinical outcomes. The development of standardised digital coordination platforms between hospital and community settings represents a priority for improving the organisational efficiency of RPM programmes.

## 5. Conclusions

This survey is among the first to assess the organisational impact of RPM for CHF management across both hospital and community practice settings in France. The results demonstrate that RPM has driven substantial organisational restructuring, with dedicated teams (96.4%), task delegation (89.3%), and specific consultations (75.0%) now widely implemented. However, significant challenges remain in professional training (53.6%), coordination with community physicians (35.7% improvement), and the adoption of digital coordination tools. As RPM transitions from experimental to standard practice in France, addressing these organisational gaps will be essential to realise the full clinical and economic potential of remote monitoring for heart failure management. The findings of this survey can serve as a practical resource for cardiology departments considering or optimising RPM implementation.

## Figures and Tables

**Table 1:** Baseline characteristics of the respondents (N = 28).

Characteristic	n	%
**Type of structure**		
Centre Hospitalier Général (CHG)	14	50.0%
Centre Hospitalier Universitaire (CHU)	9	32.1%
Community practice (cabinet de ville)	4	14.3%
Private clinic	1	3.6%
**Dedicated RPM team size**		
Mean (SD)	4.8 (2.3)	—
Median	5.0	—
Range	2–11	—
Data available (n)	25 structures	—

CHG: Centre Hospitalier Général; CHU: Centre Hospitalier Universitaire; SD: standard deviation; RPM: remote patient monitoring.

**Table 2:** Impact of RPM on the care process (N = 28).

Criterion	Yes (n)	Yes (%)	No (%)
Dedicated RPM team	27	96.4%	3.6%
Specific consultations for alerts	21	75.0%	25.0%
Direct admission protocol (bypassing ED)	16	57.1%	42.9%
Reduced time to care reported	27	96.4%	3.6%

ED: emergency department; RPM: remote patient monitoring.

**Table 3:** Delegated tasks among structures with task delegation (N = 25).

Delegated Task	n	%
Alert monitoring	25	100.0%
Patient inclusion	23	92.0%
Patient contact	21	84.0%
Treatment adjustment	12	48.0%

Proportions are calculated relative to the 25 structures with task delegation implemented.

**Table 4:** Perceived impact of RPM on quality of life and working conditions (N = 28).

Assessment	n	%
**Impact on patient quality of life**		
Very positive	18	64.3%
Positive	10	35.7%
Neutral or negative	0	0.0%
**Impact on working conditions**		
Improvement	9	32.1%
Stable	18	64.3%
Degradation	1	3.6%

RPM: remote patient monitoring.

**Table 5:** Perceived advantages and areas for improvement (N = 28, multi-response items).

Item	n	%
**Perceived advantages**		
Reduction in hospitalisations	25	89.3%
Improved patient–clinician communication	24	85.7%
Reduced time to care	21	75.0%
Reduced duration of hospitalisation	19	67.9%
**Areas for improvement**		
Interprofessional coordination	15	53.6%
Professional training	8	28.6%
Human resources allocation	6	21.4%
Dedicated time for RPM activities	2	7.1%

Multi-response items: percentages are calculated relative to the total number of respondents (N = 28) and do not sum to 100%. RPM: remote patient monitoring.

## Data Availability

The data that support the findings of this study are available from the corresponding authors upon reasonable request.

## Abbreviations

The following abbreviations are used in this manuscript:

CHF	Chronic Heart Failure
CHG	Centre Hospitalier Général
CHU	Centre Hospitalier Universitaire
ESC	European Society of Cardiology
ETAPES	Expérimentations de Télémédecine pour l’Amélioration des Parcours En Santé
HAS	Haute Autorité de Santé
HF	Heart Failure
IPA	Infirmier(ère) en Pratique Avancée
ISPIC	Infirmier(ère) Spécialisé(e)
RPM	Remote Patient Monitoring
SD	Standard Deviation
